# Five-factor personality dimensions and their associations with early maladaptive schemas in individuals with major depressive disorder

**DOI:** 10.1192/j.eurpsy.2023.521

**Published:** 2023-07-19

**Authors:** K. Koutra, G. Mavroeides, M. Basta, A. Vgontzas

**Affiliations:** ^1^Department of Psychology, University of Crete, Rethymnon; ^2^Department of Psychiatry & Behavioral Sciences, Faculty of Medicine, University of Crete; ^3^Mobile Mental Health Unit, University Hospital of Heraklion, University of Crete, Heraklion, Greece; ^4^Sleep Research & Treatment Center, Department of Psychiatry & Behavioral Health, Penn State College of Medicine, Hershey, PA, United States

## Abstract

**Introduction:**

Major depressive disorder (MDD) is the third leading cause of disease burden, accounting for 4.3% of the global burden of disease. Personality traits, as described in the Five-Factor Model, are consistently associated with individual’s well-being and mental health. Early Maladaptive Schemas (EMS) are self-perpetuating dysfunctional cognitive structures that have been linked with psychological health and play a significant role in developing and maintaining psychological distress. Both personality traits and EMS have been extensively studied as contributors to MDD symptoms.

**Objectives:**

To our knowledge, very few studies have attempted to link personality to EMS in clinical samples. The present study aimed to investigate the association between EMS with personality traits of Five-Factor Model in a clinical sample of patients with MDD in Crete, Greece.

**Methods:**

Two hundred and two patients with a clinical diagnosis of MDD (81.7% females, aged 47.75±14.06 years) participated in the study. The Traits Personality Questionnaire was used to measure personality traits in terms of neuroticism, extraversion, openness, agreeableness, and conscientiousness dimensions. The Young Schema Questionnaire-Short Form 3 (YSQ-SF 3) was used to evaluate 18 EMS which are grouped in five domains: disconnection and rejection, impaired autonomy and performance, impaired limits, other-directedness, and overvigilance and inhibition.

**Results:**

Significant associations between EMS and personality traits were found. Specifically, a higher level of all EMS domains was found in patients with MDD scoring higher in neuroticism and lower in extraversion, conscientiousness and agreeableness (apart from the association of agreeableness with other-directedness which was non-significant). Openness was negatively related to other-directedness.

**Conclusions:**

Although causal inferences cannot be made due to the cross-sectional design of the present study, our findings are in accordance with Schema Therapy that affirms a relationship between innate temperament and EMS. Future research should examine whether psychological interventions focusing at healing EMS will contribute to alteration of personality traits.

**Disclosure of Interest:**

None Declared

